# Autoimmune fibroinflammation in IgG4-related ophthalmic disease: TLR8-dependent signaling pathways and fibrotic remodeling revealed by proteomic profiling

**DOI:** 10.3389/fimmu.2026.1669568

**Published:** 2026-01-28

**Authors:** Yingxue Ma, Dejuan Song, Chuanjie Xu, GuangYu Li

**Affiliations:** 1 Department of Ophthalmology, The Second Hospital of Jilin University, Changchun, China; 2 Department of Pathology, The Second Hospital of Jilin University, Changchun, China

**Keywords:** autoimmunity, proteomics, IgG4-related disease, lacrimal gland, fibroinflammatory lesion

## Abstract

**Background:**

Immunoglobulin G4-related ophthalmic disease (IgG4-ROD) is an immune-mediated ocular condition characterized by tumefactive lesions with IgG4+ plasma cell infiltration, and commonly affecting the lacrimal gland, extraocular muscles, and trigeminal nerves.The precise pathogenesis of IgG4-ROD remains unclear. Elucidating its molecular mechanisms is crucial for the development of targeted molecular therapies.

**Methods:**

To investigate the molecular pathogenesis of IgG4-ROD, we conducted a case-controlled study involving 15 patients who presented at the Second Hospital of Jilin University between 2021 and 2022. In accordance with the comprehensive diagnostic criteria for IgG4-related disease (IgG4-RD) established in 2011, participants were stratified into three distinct cohorts based on lacrimal gland histopathological findings: confirmed IgG4-ROD, suspected IgG4-ROD, and a control group. We then utilized 4D-Fast DIA technology to acquire proteomic profiles from the lacrimal gland biopsies, with rigorous bioinformatics methodologies employed to process and interpret these data, focusing on the differential protein expression patterns across the groups, aiming to identify signaling pathways that are significantly associated with IgG4-ROD.

**Results:**

The comparative analysis of clinical characteristics, imaging features, and histopathological findings among the control group, patients with suspected IgG4-ROD, and diagnosed patients revealed a progressive trend towards more severe pathology. Principal component analysis (PCA) and Pearson correlation heatmaps indicated that the profiles of differentially expressed proteins in lacrimal gland samples from the suspected and diagnosed groups were highly similar, suggesting that the patients from these two groups may belong to the same population. Further analysis of protein expression changes across the three groups revealed significant enrichment in pathways related to asthma, Th1 and Th2 cell differentiation, and other relevant signaling pathways. Notably, Toll-like receptor (TLR), NF-κB, Wnt, and PI3K-AKT signaling pathways were prominently activated. In the diagnosed group, proteins such as MMP7, POSTN, and CD163 exhibited characteristic high-level expression. Furthermore, compared with the suspected group, the diagnosed group showed significant downregulation of proteins related to elastin fibers, indicating a more severe degree of fibrosis in the lacrimal gland tissues. Additionally, the diagnosed group exhibited a significant decrease in proteins associated with lacrimal secretion, suggesting impaired function of lacrimal gland. We also observed a notable upregulation of TLR-8-related proteins in both the suspected and diagnosed groups, implying that the TLR signaling pathway may be closely related to this disease.

**Conclusions:**

Proteomic analysis of lacrimal gland samples from the diagnosed, suspected, and control groups suggests that patients with suspected IgG4-ROD may be part of the same population as diagnosed patients. In the diagnosed group, the lacrimal gland tissue displayed more severe fibrosis and a significant loss of lacrimal secretion function. This study postulates that the TLR - 8/IRAK4/NF - κB pathway may contribute to the molecular pathogenesis of IgG4 - ROD by promoting tissue fibrosis. Proteins such as MMP7, POSTN, and CD163 could potentially serve as molecular markers for the early diagnosis and potential therapeutic targets of IgG4 - ROD. Based on these findings, proteomics offers significant advantages in the molecular diagnosis of IgG4 - ROD and should be regarded as a crucial tool in the diagnosis of this disease.

## Graphical Abstract

**Figure:**
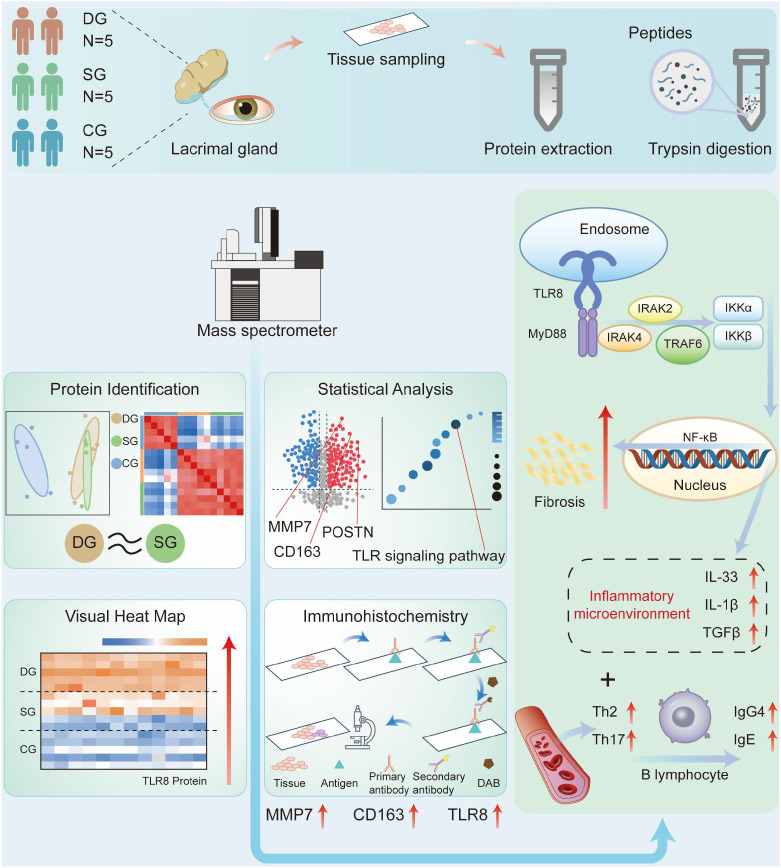

## 1 Introduction

IgG4-related disease (IgG4-RD) is an established autoimmune fibrotic inflammatory disorder. It is characterized by aberrant fibrous proliferation, widespread lymphocytic infiltration, and a conspicuous infiltration of IgG4-positive plasma cells. Epidemiologically, it is estimated that its incidence rate is approximately 0.28 to 1.08 cases per 100,000 individuals. Therefore, IgG4-RD is considered to be a relatively rare disease (1, 2). IgG4-RD can affect multiple organs, including the lacrimal glands, salivary glands, pancreas, liver, biliary tract, kidneys, lymph nodes, thyroid, and retroperitoneal region (3). The incidence is slightly higher in males than in females, with peak onset typically occurring between the ages of 50 and 70 years. If left untreated, severe cases may result in permanent organ damage or even death (4). Although most patients respond well to glucocorticoid therapy, relapse rates remain high, and there is currently an absence of effective targeted therapies for precision medicine (5).

When IgG4-RD involves the ocular region, it is termed IgG4-related ophthalmic disease (IgG4-ROD). The incidence of IgG4-ROD in the IgG4-RD population is approximately 23% (6). IgG4-ROD can affect various ocular tissues, with the lacrimal glands being the most commonly involved, followed by the extraocular muscles, eyelids, and trigeminal nerve. Clinical manifestations include painless upper eyelid swelling, lacrimal gland enlargement, proptosis, ocular motility impairment, diplopia, and vision loss secondary to optic nerve involvement (6). Some patients may also present with other organ involvement, most commonly the salivary and submandibular glands. Additionally, autoimmune pancreatitis, sclerosing cholangitis, interstitial nephritis, and retroperitoneal fibrosis are frequently observed as well (7). A history of allergic diseases, such as asthma or allergic rhinitis, may be associated with the development of IgG4-ROD (1).

To date, the pathogenesis of IgG4-ROD remains unclear. Studies have indicated that plasma cells, CD4^+^ cytotoxic T lymphocytes (CTLs), granzyme A^+^ CD8^+^ CTLs, and follicular helper T cells exhibit abnormal proliferation in affected tissues, suggesting that both innate and adaptive immune responses contribute to the disease process (8–12). Additionally, abnormal infiltration of M2 macrophages plays a key role in the fibrosis of IgG4-RD lesions, and inflammatory cytokines such as transforming growth factor-beta (TGF-β), interleukin-4 (IL-4), and interleukin-10 are implicated in promoting disease progression (13). Early diagnosis of IgG4-ROD remains a challenge. Currently, according to the consensus of IgG4-RD study groups, the primary diagnostic criteria include characteristic clinical manifestations, imaging evidence, significantly elevated serum IgG4 levels, and histopathological identification of abundant IgG4-positive plasma cells in tissue specimens (14). However, early-stage IgG4-ROD often lacks typical clinical manifestations, and serum IgG4 levels may exhibit minimal variation. In some patients, serum IgG4 levels may decrease following glucocorticoid treatment, but in cases with severe tissue fibrosis, the number of IgG4^+^ plasma cells may actually decrease upon pathological examination (15). These atypical changes further complicate the diagnostic process for IgG4-ROD. Therefore, further research into the molecular pathological mechanisms of the disease is essential to identify specific diagnostic biomarkers and facilitate early diagnosis and intervention.

Currently, there is no ideal animal model for the investigation of IgG4-ROD. However, the advent of high-throughput multi-omics technologies has provided a promising avenue to deepen our understanding of the molecular pathology underlying IgG4-ROD. These advanced methodologies have proven instrumental in elucidating the complex pathophysiological mechanisms of IgG4-ROD and facilitating the identification of novel diagnostic biomarkers. For instance, Sembler-Møller et al. utilized proteomics to analyze protein expression differences in lip and salivary gland tissue samples from IgG4-RD and Sjögren’s syndrome (SS) patients, identifying potential diagnostic biomarkers such as neutrophil elastase, calmodulin, and tripartite motif proteins. Their findings suggest that protein expression levels in saliva could serve as a potential diagnostic tool for primary SS (16). Additionally, Zhang Yusheng and colleagues employed isotope-labeled relative and absolute quantification proteomics to analyze serum and submandibular gland tissue samples from IgG4-RD patients and healthy controls. They found extensive IgG4 plasma cell infiltration in the submandibular glands, particularly in samples with severe tissue fibrosis, where the expression levels of cytochrome C, somatic (CYCS), reactive oxygen species (ROS), and integrin family proteins were significantly elevated (17).

In this study, we conducted a case-control investigation utilizing high-throughput proteomics technology to analyze protein expression levels in lacrimal gland samples of varying severity from patients with IgG4-ROD. By comparing protein expression profiles between normal lacrimal glands, lacrimal glands of suspected IgG4-ROD, and histopathologically diagnosed IgG4-ROD, our objective is to identify the autoimmune pathways of IgG4-ROD dysregulation and novel diagnostic biomarkers and uncover the molecular drivers of fibrosis. This effort aims to lay the groundwork for elucidating the molecular pathological mechanisms underlying the disease. Ultimately, these findings could contribute to the development of effective, specific molecular-targeted therapies for IgG4-ROD.

## 2 Methods

### 2.1 Sample collection and grouping criteria

Although multiple international expert consensuses on the diagnosis and treatment of IgG4-RD have been published in recent years, for cases of IgG4-ROD that primarily present in or are solely limited to the ocular, the IgG4-RD diagnosis and treatment consensus published by the Umehara and Okazaki study groups in Japan in 2011 is more practical and operationally feasible in clinical applications. Therefore, this study mainly refers to this expert consensus for grouped research.

This study employs a case-control design and recruited a total of 15 subjects from January 2021 to January 2022 at the Second Hospital of Jilin University. Due to the epidemiological rarity of IgG4-ROD, there are limitations in obtaining a large number of clinical samples. Based on the inclusion criteria, these subjects were stratified into three groups: diagnosed group (n=5), suspected group (n=5), and control group (n=5). Lacrimal gland samples were collected from all groups. None of the patients in the diagnosed group received glucocorticoid therapy.

#### 2.1.1 Inclusion criteria

Diagnosed Group: Participants in the diagnosed group met the comprehensive diagnostic criteria for IgG4-RD established by the Umehara and Okazaki study groups in 2011 and the diagnostic criteria for IgG4-ROD in 2015 (18, 19). These criteria included:

1. Imaging studies show enlargement of the lacrimal gland, trigeminal nerve, or extraocular muscle as well as masses, enlargement, or hypertrophic lesions in various ophthalmic tissues.
2. Histopathologic examination shows marked lymphocyte and plasmacyte infiltration, and sometimes fibrosis. A germinal center is frequently observed. IgG4+ plasmacytes are found and satisfy the following criteria: ratio of IgG4+ cells to IgG+ cells of 40% or above, or more than 50 IgG4+ cells per high-power field (×400).
3. Blood test shows elevated serum IgG4 (≥135 mg/dl).

Patients who meet all three criteria (1, 2, and 3) are classified as the diagnosed group.

Suspected Group: We classified patients who meet any two of the criteria of the diagnosed group as the suspected group. Patients in the suspected group presented with palpable enlargement of the lacrimal gland tissue in the upper orbit. Although their lacrimal gland samples showed no significant differences from those of the diagnosed group under hematoxylin and eosin (HE) staining, immunohistochemical results revealed scattered IgG4^+^ plasma cells, with a count significantly lower than the threshold required for the diagnosis of IgG4-RD.

Control Group: The subjects in the control group had lacrimal gland prolapse, but no clinical manifestations such as extraocular muscle thickening, eyelid swelling, proptosis or pain occurred. To further rule out non-specific inflammation of the lacrimal gland, we removed a small portion of the lacrimal gland for biopsy. The pathological examination results showed no significant abnormal changes. Therefore, we defined it as non-specific tear gland prolapse and included it in the control group.

#### 2.1.2 Exclusion criteria

Patients with other conditions were excluded from the study:

- Other inflammatory lacrimal gland lesions or lacrimal gland tumors (such as lacrimal gland lymphoma).
- Other autoimmune diseases.
- Active or severe infections.
- Malignancies.

### 2.2 Protein extraction

After deparaffinization, the samples were transferred to 1.5 ml centrifuge tubes, and four times the volume of lysis buffer (1% SDS Lysis Buffer, 1% protease inhibitor) was added, followed by ultrasonication. The samples were then centrifuged at 4 °C, 12,000 g for 10 minutes to remove cellular debris, and the supernatant was collected and transferred to new centrifuge tubes. Finally, protein concentration was determined using a Bicinchoninic Acid Assay (BCA) protein assay kit.

### 2.3 Trypsin digestion

Equal amounts of protein from each sample were taken for digestion. After adjusting the volume with lysis buffer to match, 1 volume of pre-chilled acetone was added, and the samples were vortexed. Then, 4 volumes of pre-chilled acetone were added, and the samples were precipitated at -20 °C for 2 hours. The samples were centrifuged at 4500 g for 5 minutes, and the supernatant was discarded. The pellet was washed 2–3 times with pre-chilled acetone and air-dried. The pellet was resuspended in TEAB to a final concentration of 200 mM, and ultrasonication was applied to disperse the pellet. Trypsin was added at a ratio of 1:50 (trypsin:protein, w/w), and digestion was carried out overnight. Dithiothreitol was added to a final concentration of 5 mM, and the samples were reduced at 56 °C for 30 minutes. Subsequently, iodoacetamide was added to a final concentration of 11 mM, and the samples were incubated in the dark at room temperature for 15 minutes.

### 2.4 Liquid chromatography-mass spectrometry analysis

The peptides were dissolved in mobile phase A for liquid chromatography separation using the Vanquish ultra-high-performance liquid chromatography system. Mobile phase A consisted of an aqueous solution containing 0.1% formic acid and 2% acetonitrile, while mobile phase B consisted of an aqueous solution containing 0.1% formic acid and 90% acetonitrile. The liquid chromatography gradient was set as follows: 0-22.5 min, 6%-22% B; 22.5-26.5 min, 22%-34% B; 26.5-28.5 min, 34%-80% B; 28.5–30 min, 80% B, with a flow rate maintained at 700 nl/min. After separation by the ultra-high-performance liquid chromatography system, the peptides were injected into the NSI ion source for ionization and then analyzed by Orbitrap Exploris 480 mass spectrometry. The ion source voltage was set to 2300 V, and the High-field asymmetric waveform ion mobility spectrometry compensation voltage was not specified. The peptide precursor ions and their secondary fragments were detected using high-resolution Orbitrap. The scan range for the first MS was set to 350–1400 m/z with a resolution of 60,000, and the scan range for the second MS was set with a fixed starting point at 120 m/z and a resolution of 15,000. The data acquisition mode was data-independent acquisition (DIA), where, after the first scan, ions from multiple consecutive m/z windows entered the highly conserved domain collision cell, fragmented with 27% collision energy, and subsequently underwent second-stage mass spectrometry analysis. To improve mass spectrometry efficiency, automatic gain control was set to 1E6, and the maximum injection time was set to 22 ms.

### 2.5 Protein identification and quantification

The DIA data were analyzed using the DIA-NN (v 1.8) search engine. The database used was Homo_sapiens_9606_SP_20230103.fasta (20,389 sequences), and digestion was performed using Trypsin/P with a maximum of one allowable missed cleavage. Fixed modifications included N-terminal methylation (N-term M excision) and C-terminal carbamylation (C carbamidomethylation). A theoretical spectral library was constructed using deep learning algorithms, and a decoy database was included to calculate the false discovery rate (FDR) caused by random matches. The FDR for precursor identification was set to 1%.

### 2.6 Immunohistochemical analysis

The immunohistochemical staining was performed using the Strept Avidin-Biotin Complex (SABC) method. After the tissues were fixed with 4% paraformaldehyde, 4-μm paraffin sections were prepared. Following dewaxing and antigen retrieval, the endogenous peroxidase was blocked with 3% hydrogen peroxide. The primary antibody was added and incubated overnight at 4 °C, followed by sequential incubation with the secondary antibody and the SABC complex. The color development was carried out using a diaminobenzidine kit, and counterstaining was performed with hematoxylin. After dehydration with gradient ethanol, clearing with xylene, the sections were sealed with neutral balsam. Microphotographs were obtained using an optical microscope equipped with a digital camera. The immunohistochemical staining results were evaluated based on predefined color and morphological characteristics: no cellular staining was considered negative; light yellow fine granules were classified as weakly positive; brownish-yellow granules were regarded as moderately positive; and dark brown coarse granules were identified as strongly positive. For quantitative evaluation, Image J software was utilized to calculate the percentage of positive area relative to the total analyzed region, thereby quantifying the expression level of the target protein.

### 2.7 Statistical analysis

Statistical analysis was performed using one-way ANOVA to compare differences in immunohistochemical signal intensity among groups, with a p-value<0.05 considered statistically significant. The corresponding column graph was generated using GraphPad Prism 10.1. Statistical analysis was carried out to identify differentially expressed proteins (DEPs) from the proteomic datasets. Following log2 transformation and quantile normalization of protein abundance values, DEPs were assessed using Student’s t-test and ANOVA. Proteins with a p-value<0.05 and a fold change ≥1.5 (up-regulated) or ≤0.67 (down-regulated) were considered significant. Volcano plots and bar charts were generated using GraphPad Prism 10.1 for data analysis and figure creation. Visual bioinformatics tools (https://www.bioinformatics.com.cn) were used to perform protein functional enrichment analysis using Gene Ontology (GO), Kyoto Encyclopedia of Genes and Genomes (KEGG), and Reactome databases, and bubble charts were generated. Additionally, a heatmap was generated to visualize the data matrix of protein expression levels.

## 3 Results

### 3.1 General demographic information and clinical features

In this study, a total of 15 participants were recruited. The subjects were classified into three groups according to the inclusion criteria: the diagnosed group, the suspected group, and the control group, with five subjects in each group. The participants in all three groups were middle-aged, with an average age of 45.67 ± 7.93 years. There were no significant statistical differences in terms of age and gender among the subjects in the three groups.

Clinically, the patients in both diagnosed and suspected group exhibited eyelid swelling, with enlarged lacrimal gland tissue palpable above the orbit, which was firm and painless in both groups. The patients in diagnosed group also presented with exophthalmos, and four cases exhibited associated ocular motility disorders. In contrast, the participants in control group showed no lacrimal gland enlargement or other ocular symptoms.

**Figure 1:**
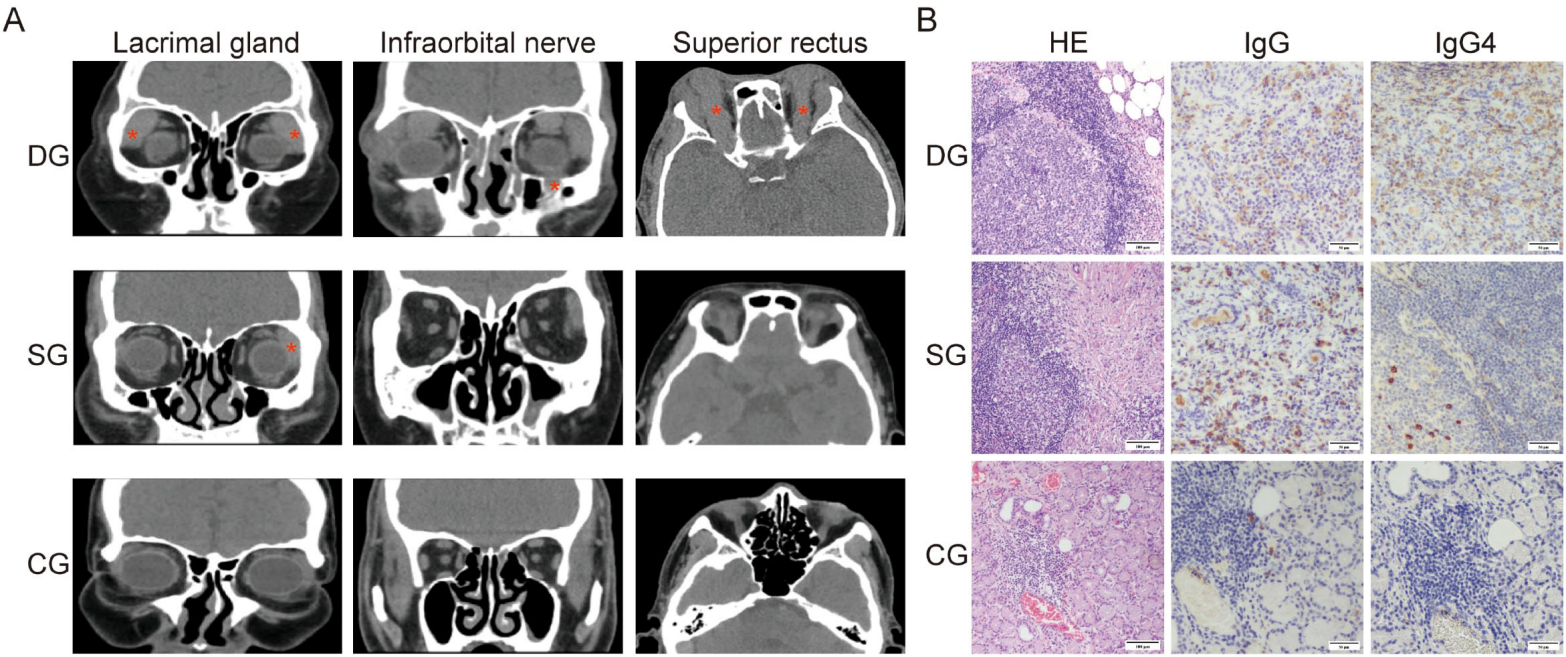
Imaging features and lacrimal gland pathological manifestations in IgG4-ROD diagnosed/suspected patients. **(A)** Orbital CT Coronal and Horizontal Views: In the diagnosed group, bilateral lacrimal gland enlargement is observed, with substantial thickening of the superior rectus muscle and infraorbital nerve (*), accompanied by inflammation-like changes of sinus. In the suspected group, unilateral lacrimal gland enlargement is noted (*). **(B)** Hematoxylin and Eosin (HE) Staining of Lacrimal Gland: In both the diagnosed and suspected groups, lacrimal gland tissue exhibits diffuse infiltration by a large number of lymphocytes and plasma cells, with numerous lymphoid follicle formations. The stromal fibrous tissue demonstrates varying degrees of proliferation (scale bar: 100 μm). Immunohistochemical Staining: In the diagnosed group, the number of IgG4^+^ plasma cells exceeds 50 per high-power field; in the suspected group, less than 10 IgG4^+^ plasma cells are observed per high-power field; in the control group, no or only a very small number of scattered IgG4^+^ plasma cells are present (scale bar: 50 μm). DG, Diagnosed group; SG, Suspected group; CG, Control group.

In terms of imaging findings, there were distinct differences in orbital CT scans among the three groups (**Figure 1A**). CT scans of patients in the diagnosed group consistently revealed symmetric, enlarged nodular soft tissue density in the bilateral lacrimal gland regions, which presented with uniform density and indistinct borders merging with the adjacent extraocular muscles. Some of the extraocular muscles and the infraorbital nerve were thickened, and diffuse abnormalities were detected within the orbit. No abnormalities were noted in the size or shape of the bilateral eyeballs. Imaging in three cases of the diagnosed group indicated varying degrees of unilateral or bilateral inflammatory changes in the frontal, ethmoid, maxillary, and sphenoid sinuses. One case exhibited bilateral submandibular gland echogenicity abnormalities accompanied by concurrent internal lymphadenopathy. In the suspected group, imaging predominantly showed unilateral or bilateral enlargement of the lacrimal glands, with one patient displaying inflammatory changes in the paranasal sinuses. The control group demonstrated no abnormal findings on orbital CT.

Histopathological examination of lacrimal gland tissue specimens served as the primary basis for patient stratification. HE staining revealed extensive lymphoplasmacytic infiltration with prominent lymphoid follicle formation in both the diagnosed and suspected IgG4-ROD groups. The degree of eosinophil infiltration varied among cases, accompanied by varying extents of stromal fibrosis. Obstructive phlebitis was observed in a subset of specimens. In contrast, control group tissues maintained normal lacrimal gland architecture with only minimal chronic inflammatory cell infiltration. Immunohistochemical analysis demonstrated that in the diagnosed group, IgG4^+^ plasma cell counts exceeded 50 per high-power field (HPF), with certain cases surpassing 200/HPF, while the IgG4^+^/IgG^+^ plasma cell ratio was greater than 40%. The suspected group exhibited fewer than 10 IgG4^+^ plasma cells per HPF and an IgG4^+^/IgG^+^ ratio below 40%. Control samples showed either absence or only sporadic presence of IgG4^+^ plasma cells (**Figure 1B**).

Overall, from the suspected group to the diagnosed group, patients manifested progressively more prominent clinical symptoms, a wider range of imaging lesions, and an increasing number of IgG4^+^ plasma cells and fibrous tissue proliferation in the pathological examination. Integrating clinical, radiological, and histopathological data from lacrimal gland tissue thus allows for the definition of IgG4-ROD severity, wherein the diagnosed group is characterized by a more severe disease state compared to the suspected group.

### 3.2 Proteomic analysis and protein identification

**Figure 2:**
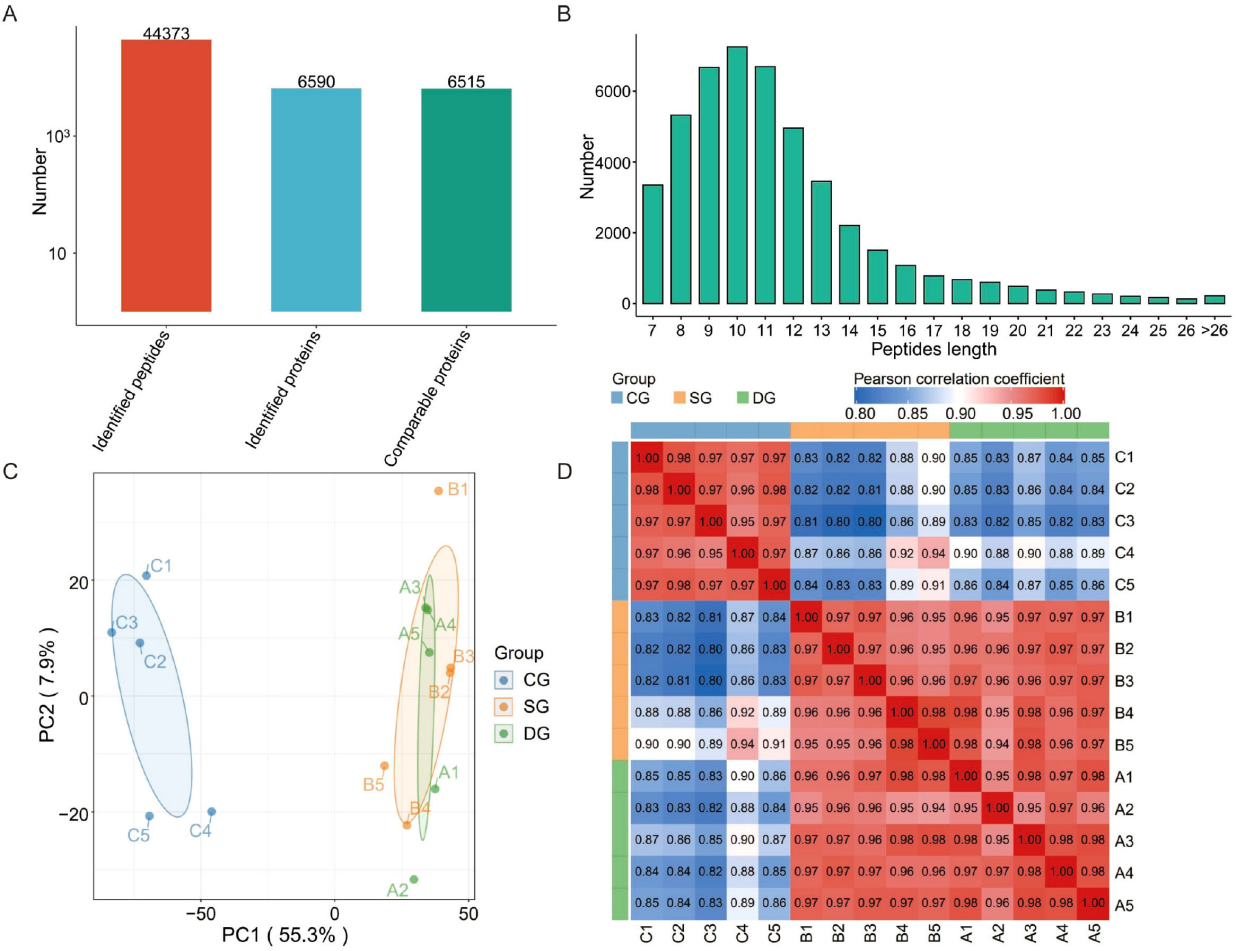
Proteomic analysis of lacrimal glands in the IgG4-ROD diagnosed, suspected, and control groups. **(A)** Outcomes of mass spectrometry analysis, illustrating the quantities of peptides, proteins, and comparable proteins acquired. **(B)** Distribution graph of peptide length and amount derived from mass spectrometry analysis. **(C)** Principal Component Analysis (PCA) of protein expression in lacrimal gland samples from the diagnosed, suspected, and control groups. Notably, circles of diverse colors mirror the clustering of samples from distinct groups, while the size of the circle denotes the degree of dispersion within the respective group. **(D)** Visual heatmap of protein expression in lacrimal gland samples from the diagnosed, suspected, and control groups. This map is predicated on the Pearson correlation coefficient, wherein a darker red hue signifies a stronger correlation and a darker blue shade indicates a weaker correlation. DG, Diagnosed group; SG, Suspected group; CG, Control group.

Employing DIA technology, proteomic data were derived from the lacrimal gland samples of 15 participants. This yielded 44,373 peptide sequences and 6,590 proteins. A total of 6,515 proteins were identified as suitable for subsequent comparative analysis. The distribution of peptide lengths and their respective numbers are presented in **Figures 2A, B**. The results manifested a considerable variance in peptide lengths, which is indicative of high-quality mass spectrometry data. Such quality is advantageous for further in-depth analysis. Principal Component Analysis (PCA) unveiled substantial disparities in protein expression levels and clustering patterns among the lacrimal gland samples from the three groups (**Figure 2C**). As depicted in the figure, the lacrimal gland samples from the diagnosed and suspected groups were clearly segregated from those of the control group. This indicates significant differences among the three groups, suggesting that the samples were sourced from distinct populations. Moreover, the protein expression profiles of the diagnosed and suspected groups were remarkably similar, implying that these two groups might belong to the same population. Within each group, the samples clustered closely, demonstrating strong internal consistency. **Figure 2D** showcases a heatmap constructed based on Pearson correlation coefficients, where the intensity of color represents the degree of correlation between samples. A deeper red color corresponds to a stronger correlation, while a deeper blue color indicates a weaker correlation. The p-values within each of the three groups were highly similar, presenting as a deep red color, signifying robust within-group correlations. In contrast, the p-values between groups exhibited notable differences, appearing as blue or light red. Thus, in line with the PCA analysis, this suggests significant differences among the three groups, with the diagnosed and suspected groups showing a greater degree of similarity, intimating that they may be part of the same population. Preliminary assessment revealed robust reproducibility within each experimental group, indicating high sample homogeneity and representativeness. Concurrently, we observed pronounced intergroup differential expression of proteins. Subsequent investigations will, therefore, focus on elucidating the underlying signaling pathways and molecular mechanisms.

### 3.3 Comparative analysis of differential protein expression in lacrimal gland samples between the IgG4-ROD diagnosed group and the control group

**Figure 3:**
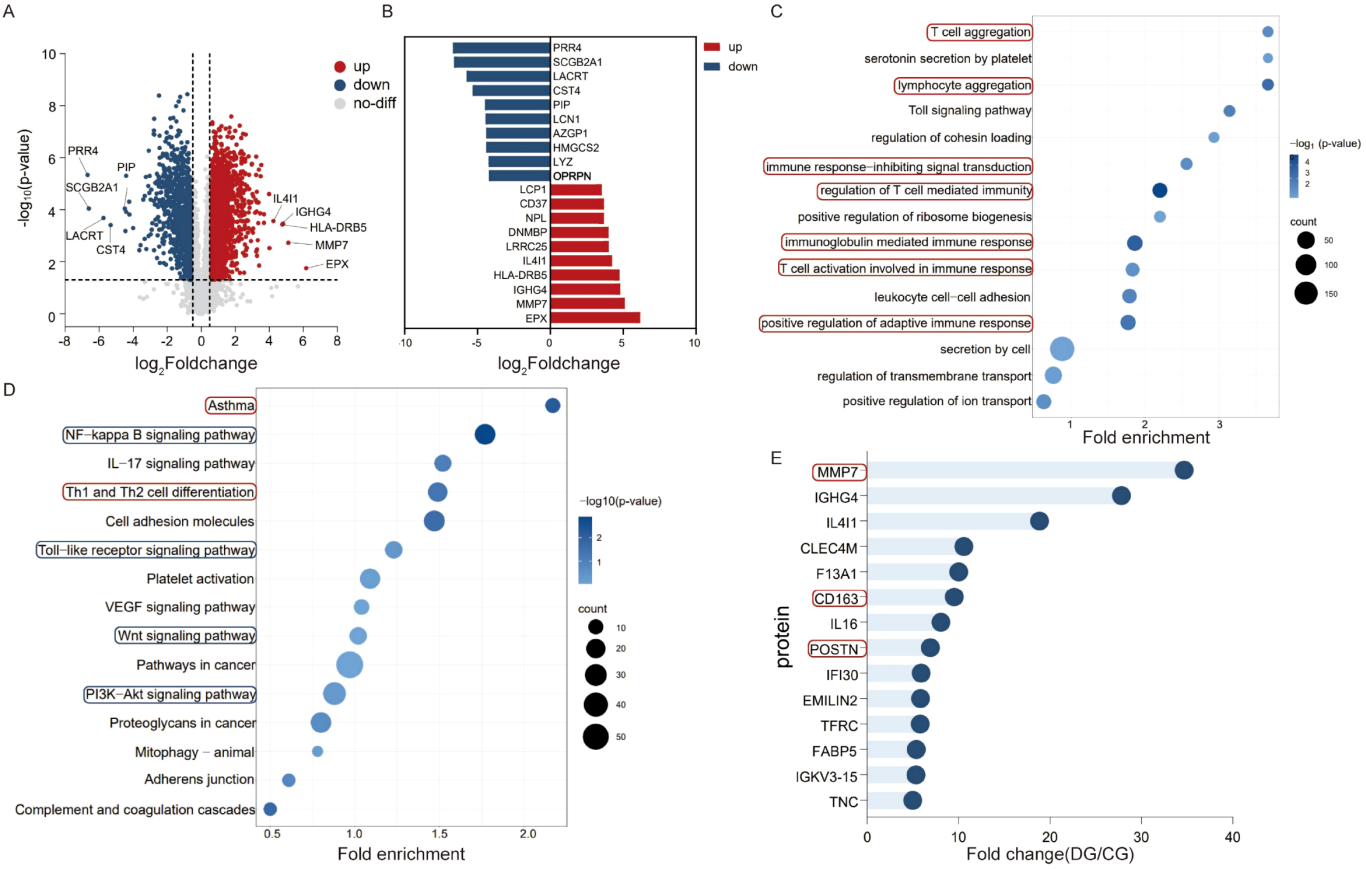
Differential expression and functional enrichment analysis of lacrimal gland samples between the diagnosed group and the control group in IgG4-ROD. **(A)** Volcano Plot of Differentially Expressed Proteins: A volcano plot was constructed to visualize differentially expressed proteins. Proteins with a fold-change greater than 2 and a P-value less than 0.05 were considered significantly differentially expressed. In this plot, upregulated proteins are denoted in red, while downregulated proteins are represented in blue. **(B)** Bar Chart of the Top Ten Differentially Expressed Proteins: A bar chart is presented to display the top ten proteins with the most significant upregulation (depicted in red) and downregulation (depicted in blue) in the lacrimal gland samples from the diagnosed group. This chart provides a clear overview of the proteins that exhibit the most pronounced differential expression between the two groups. **(C)** GO-Database-Based Functional Enrichment Analysis Bubble Chart: A bubble chart was generated to illustrate the functional enrichment analysis of differentially expressed proteins using the Gene Ontology (GO) database. The red-boxed areas highlight the immune-response pathways that are associated with the differentially expressed proteins. The size of the bubbles represents the number of genes involved in each pathway, and the color intensity corresponds to the significance level (smaller p-values are indicated by darker colors), thus allowing for easy identification of pathways that are significantly enriched. **(D)** KEGG-Database-Based Functional Enrichment Analysis Bubble Chart: This bubble chart represents the functional enrichment analysis of differentially expressed proteins based on the Kyoto Encyclopedia of Genes and Genomes (KEGG) database. The red-boxed areas highlight the immune-response pathways related to the differentially expressed proteins. Additionally, the blue-boxed areas emphasize the signal pathways that show stronger correlations with the differential expression. Similar to the GO-based bubble chart, the bubble size indicates the number of genes in each pathway, and the color represents the significance level of enrichment. **(E)** 14 Significantly Upregulated Secretory Proteins: Fourteen secretory proteins that are significantly upregulated in the lacrimal gland samples are shown. The secretory proteins marked with red boxes are hypothesized to potentially serve as molecular biomarkers for lacrimal gland fibrosis, which may play a crucial role in the early detection and understanding of the disease progression in IgG4-ROD.

Via proteomics analysis, a cumulative total of 1341 differentially expressed proteins were identified in the lacrimal gland samples of the diagnosed group when compared to those of the control group. Of these, 848 proteins manifested upregulated expression, whereas 493 proteins exhibited downregulated expression (**Figure 3A**). The top ten proteins with the most significant upregulation and downregulation are presented in **Figure 3B**. The GO functional enrichment analysis disclosed that multiple biological processes, including T cell aggregation, lymphocyte aggregation, T cell-mediated immune regulation, and immunoglobulin-mediated immune response, were activated within the diagnosed group. In the graphical illustration, these processes are indicated by darker bubble colors, which correspond to smaller p-values, thereby strongly suggesting their association with IgG4-ROD (**Figure 3C**). Furthermore, the KEGG pathway enrichment analysis demonstrated that pathways related to asthma, as well as T helper 1 (Th1) and T helper 2 (Th2) cell differentiation, were notably activated, as evidenced by darker bubble hues, hinting at their potential roles in the pathogenesis of IgG4-ROD. Notably, the bubbles representing the nuclear factor-kappaB (NF-κB) signaling pathway, IL-17 signaling pathway, Toll-like receptor signaling pathway, Wnt signaling pathway, and phosphatidylinositol 3 kinase-protein kinase B (PI3K-AKT) pathway were both larger and darker, further substantiating a robust correlation with IgG4-ROD (**Figure 3D**). Moreover, 14 upregulated secreted proteins, such as Matrix Metalloproteinase 7 (MMP7), immunoglobulin heavy constant gamma 4 (IGHG4), and IL4I1, hold potential as prospective serum biomarkers for the early detection of IgG4-ROD (**Figure 3E**).

### 3.4 Proteomic profiling reveals similar immune signaling pathway activation in suspected and diagnosed IgG4-ROD groups

**Figure 4:**
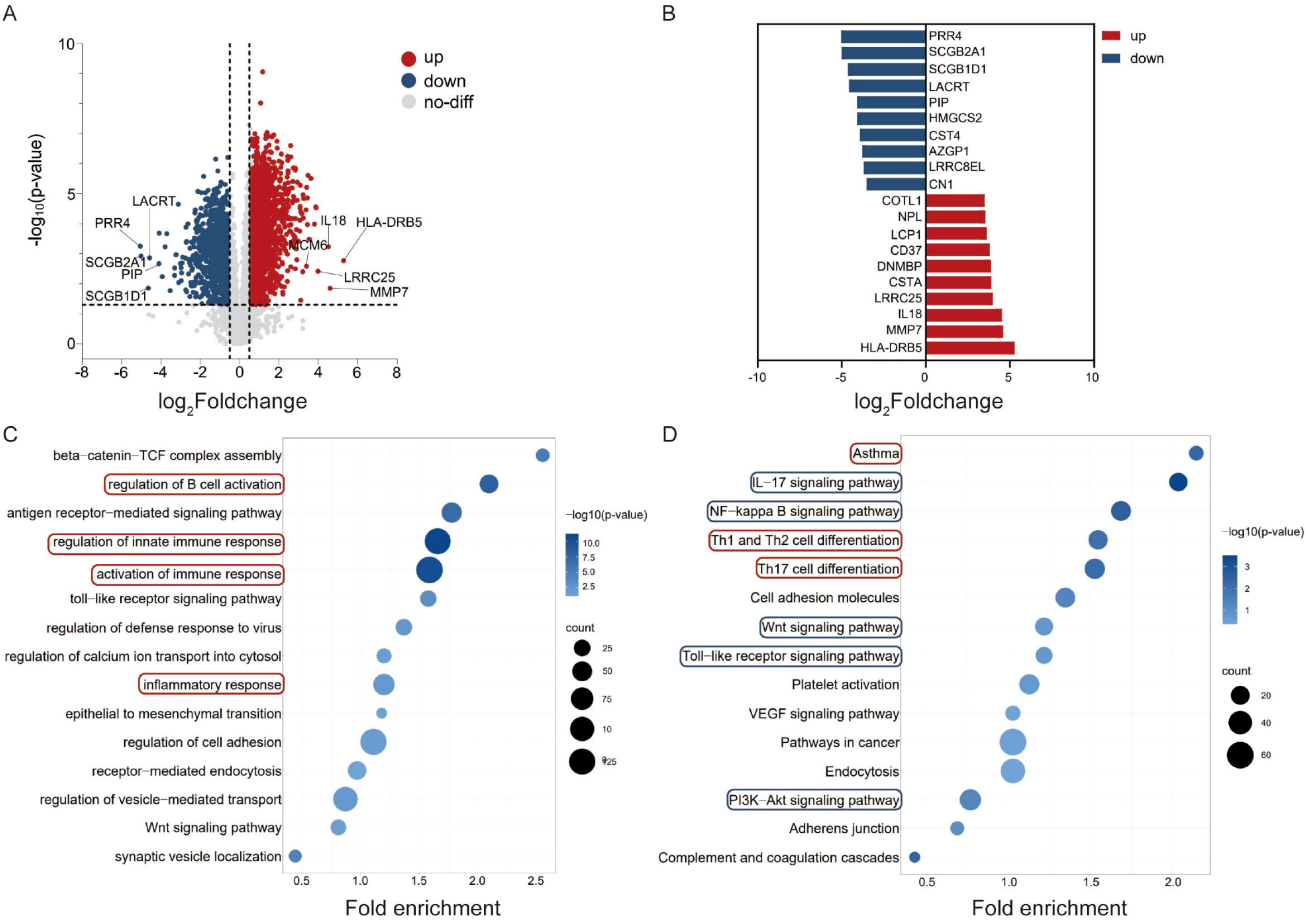
Differential expression and functional enrichment analysis of lacrimal gland samples between the suspected group and the control group in IgG4-ROD. **(A)** Volcano Plot of Differentially Expressed Proteins: A volcano plot was constructed to visualize differentially expressed proteins. Proteins with a fold-change greater than 2 and a P-value less than 0.05 were considered significantly differentially expressed. In this plot, upregulated proteins are denoted in red, while downregulated proteins are represented in blue. **(B)** Bar Chart of the Top Ten Differentially Expressed Proteins: A bar chart is presented to display the top ten proteins with the most significant upregulation (depicted in red) and downregulation (depicted in blue) in the lacrimal gland samples from the diagnosed group. This chart provides a clear overview of the proteins that exhibit the most pronounced differential expression between the two groups. **(C)** GO-Database-Based Functional Enrichment Analysis Bubble Chart: A bubble chart was generated to illustrate the functional enrichment analysis of differentially expressed proteins using the Gene Ontology (GO) database. The red-boxed areas highlight the immune-response pathways that are associated with the differentially expressed proteins. The size of the bubbles represents the number of genes involved in each pathway, and the color intensity corresponds to the significance level (smaller p-values are indicated by darker colors), thus allowing for easy identification of pathways that are significantly enriched. **(D)** KEGG-Database-Based Functional Enrichment Analysis Bubble Chart: This bubble chart represents the functional enrichment analysis of differentially expressed proteins based on the Kyoto Encyclopedia of Genes and Genomes (KEGG) database. The red-boxed areas highlight the immune-response pathways related to the differentially expressed proteins. Additionally, the blue-boxed areas emphasize the signal pathways that show stronger correlations with the differential expression. Similar to the GO-based bubble chart, the bubble size indicates the number of genes in each pathway, and the color represents the significance level of enrichment.

In comparison to the control group, we identified 1309 differentially expressed proteins in lacrimal gland samples from the IgG4-ROD suspected group, including 852 upregulated and 457 downregulated proteins (**Figures 4A, B**). GO functional enrichment analysis revealed that biological processes, including B cell activation, innate immune response, immune response activation, and inflammatory response, were significantly enriched, as indicated by larger bubble areas and darker colors, suggesting that the differentially expressed proteins were primarily enriched in immune regulation-related biological processes (**Figure 4C**). KEGG pathway enrichment analysis revealed that pathways associated with asthma, as well as Th1, Th2, and T helper 17 (Th17) cell differentiation, were enriched, as indicated by larger bubble areas, suggesting a higher number of proteins involved in these pathways. Specifically, upregulated proteins were strongly associated with the interleukin-17 (IL-17) signaling pathway, NF-κB signaling pathway, Wnt signaling pathway, Toll-like receptor signaling pathway, and PI3K-AKT signaling pathway (**Figure 4D**). The functional enrichment results for the suspected group were similar to those for the diagnosed group, with both showing activation of immune response-related biological processes and enrichment in similar signaling pathways. Therefore, further investigation is needed to explore the differences in protein expression between lacrimal gland samples from the diagnosed and suspected groups, with the aim of revealing distinctions between them.

### 3.5 Comparative Analysis of Differential Protein Expression in Lacrimal Gland Samples Between IgG4-ROD Diagnosed Group and Suspected Group

**Figure 5:**
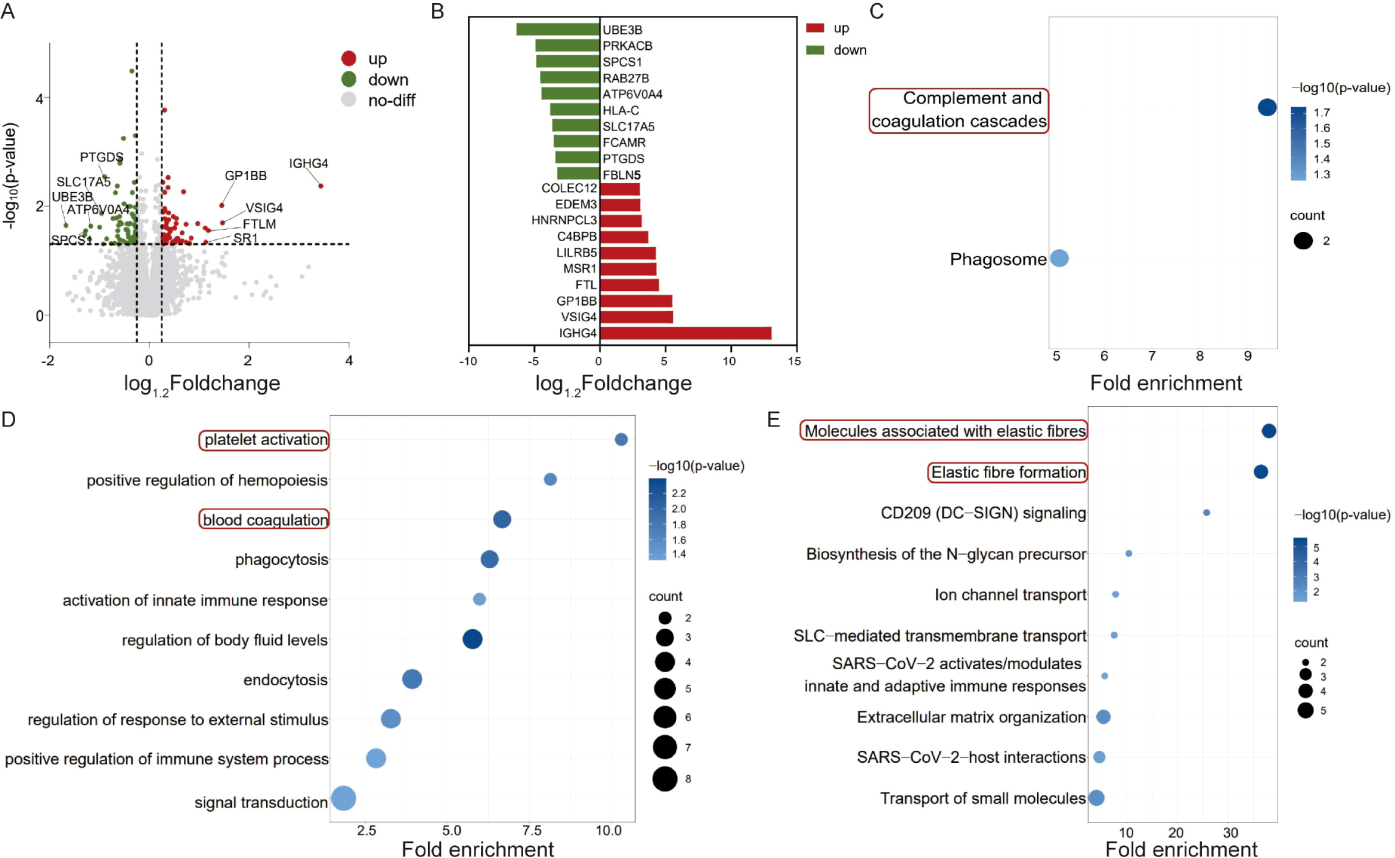
Differentially expressed proteins and functional enrichment analysis of lacrimal gland samples between the diagnosed and suspected IgG4-ROD groups. **(A)** Volcano Plot of Differentially Expressed Proteins: A volcano plot was constructed to visualize the landscape of differentially expressed proteins between the diagnosed and suspected IgG4-ROD groups. Proteins were selected according to a fold-change greater than 1.2 and a P-value less than 0.05 as criteria for significant differential expression. In this graphical representation, upregulated proteins are denoted in red, while downregulated proteins are indicated in green. This plot allows for a clear discrimination of proteins that exhibit significant expression level alterations, providing a foundation for further exploration of the molecular differences underlying the two groups. **(B)** Bar Chart of the Top Ten Differentially Expressed Proteins: A bar chart is presented to display the top ten proteins with the most prominent upregulation (depicted in red) and downregulation (depicted in green) within the lacrimal gland samples of the diagnosed group. This chart offers a concise and intuitive overview of the key proteins that drive the differential expression patterns, enabling researchers to quickly identify the most significantly affected molecular entities. **(C)** KEGG-Database-Based Functional Enrichment Analysis Bubble Plot: A bubble plot was generated to conduct a functional enrichment analysis of the differentially expressed proteins using the Kyoto Encyclopedia of Genes and Genomes (KEGG) database. The red-boxed regions indicate the complement and coagulation cascade pathways, which are strongly associated with the pathophysiology of IgG4-ROD. The size of each bubble in the plot corresponds to the number of genes involved in a particular pathway, and the color intensity represents the significance level of enrichment. Through this visualization, it becomes evident that these pathways are significantly perturbed in the context of the differential protein expression between the two groups. **(D)** GO-Database-Based Functional Enrichment Analysis Bubble Plot: This bubble plot represents the functional enrichment analysis of differentially expressed proteins based on the Gene Ontology (GO) database. The red-boxed areas highlight the active involvement of differentially expressed proteins in platelet activation and coagulation pathways. Similar to the KEGG-based bubble plot, the bubble size indicates the gene count within each pathway, and the color scale reflects the significance of enrichment. The activation of these biological processes suggests a potential role of platelet-related immune regulation in the development and progression of IgG4-ROD. **(E)** Reactome-Database-Based Functional Enrichment Analysis Bubble Plot: A bubble plot was created to perform a functional enrichment analysis of differentially expressed proteins using the Reactome database. The red-boxed regions indicate the elastic fiber-related signaling pathways, which are hypothesized to be involved in the disease progression of IgG4-ROD. The graphical representation of the bubble plot, with bubble size representing gene count and color intensity denoting enrichment significance, provides insights into how the downregulation of proteins related to these pathways may contribute to the observed differences between the diagnosed and suspected groups, potentially implicating a role in tissue remodeling and fibrosis processes associated with the disease.

In light of the substantial similarity in protein expression profiles between the diagnosed and suspected groups, a fold-change threshold of > 1.2 was applied to the disparities in protein expression. Consequently, when compared with the suspected group, the lacrimal gland tissue of the diagnosed group demonstrated 64 upregulated proteins and 70 downregulated proteins (**Figures 5A, B**). The Kyoto Encyclopedia of Genes and Genomes (KEGG) pathway enrichment analysis disclosed that the upregulated proteins were associated with the complement and coagulation cascades signaling pathways (**Figure 5C**). The GO functional enrichment analysis unveiled the activation of biological processes such as platelet activation and blood coagulation (**Figure 5D**). This finding implies that platelet-mediated immune regulation may play a role in the pathogenesis of IgG4-ROD. Furthermore, the Reactome pathway analysis revealed that the significantly downregulated proteins were enriched in elastin fiber-related signaling pathways (**Figure 5E**). The decrease in elastin fibers is a characteristic trait of tissue fibrosis. This suggests that the degree of fibrosis in the diagnosed group is more severe than that in the suspected group.

### 3.6 Differential protein expression analysis and functional enrichment analysis of lacrimal gland samples among three groups

**Figure 6:**
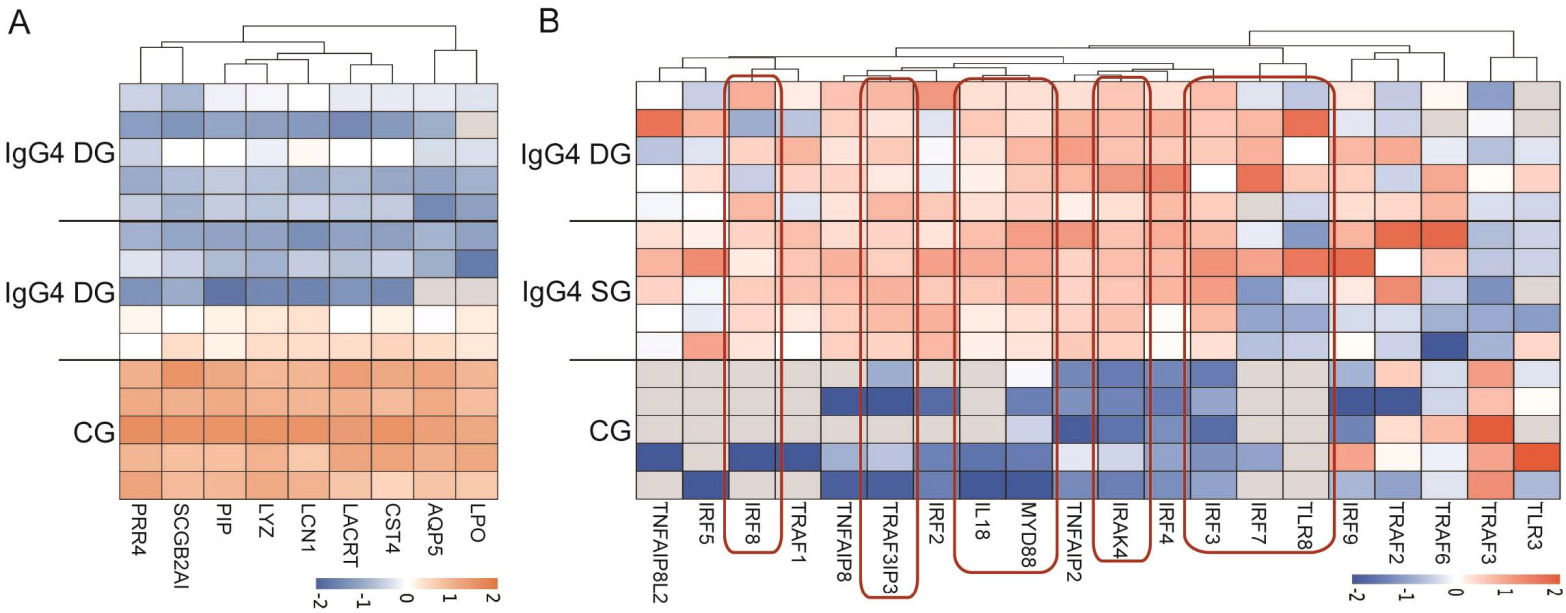
Analysis of the expression levels of lacrimal gland secretion-related proteins and TLR signaling pathway-related proteins. **(A)** Visualization Heatmap of the Expression Levels of Lacrimal Gland Secretion-Related Proteins in Lacrimal Gland Samples from the Diagnosed, Suspected, and Control Groups. This heatmap was designed to visualize the expression levels of proteins related to lacrimal gland secretion within the lacrimal gland samples obtained from the diagnosed, suspected, and control groups. In this graphical representation, red color is employed to denote upregulated expression, while blue color indicates downregulated expression. Moreover, the intensity of the color, whether red or blue, is proportional to the significance of the change in expression levels. That is to say, the darker the color, the more pronounced the alteration in the expression of the corresponding protein. Through this heatmap, one can readily observe and compare the variations in the expression of these secretion-related proteins among the different groups, thereby facilitating an understanding of the potential differences in lacrimal secretion function. **(B)** Visualization Heatmap of the Expression Levels of TLR Signaling Pathway-Related Proteins. The heatmap presented here is utilized to display the expression levels of proteins associated with the TLR signaling pathway. Similar to the previous heatmap, red color is used to signify upregulated expression and blue color represents downregulated expression. Additionally, the darker the color, the more significant the change in expression. Notably, the proteins related to the TLR8 signaling pathway are highlighted by red boxes within this heatmap. This visual aid enables researchers to quickly identify the proteins involved in the TLR8 signaling pathway and assess their expression patterns. It also helps in discerning the potential activation or suppression of the TLR signaling pathway, which may offer valuable insights into the molecular mechanisms underlying the disease under study.

Clinically, the physical signs and symptoms of patients in the suspected and diagnosed groups become increasingly prominent. This indicates that these groups may represent different stages or levels of disease severity. Through analyzing the differences in protein expression among these three groups, our objective is to identify the signaling pathways implicated in the disease pathogenesis. We also investigated the expression changes of proteins involved in lacrimal gland secretion. Results showed no significant differences between the diagnosed and suspected groups. Nevertheless, when compared with the lacrimal tissue of the control group, the expression levels of proteins related to vesicle synthesis and transport were significantly lower in the lacrimal tissue of both the diagnosed and suspected groups. This finding suggests a decline in lacrimal secretion function (**Figure 6A**). Furthermore, we observed that eight proteins associated with the Toll-like receptor (TLR) signaling pathway, namely TLR8, myeloid differentiation primary response 88 (Myd88), interleukin-1 receptor-associated kinase 4 (IRAK4), interferon regulatory factor 7 (IRF7), interferon regulatory factor 3 (IRF3), TRAF3 Interacting Protein 3 (TRAF3IP3), interferon regulatory factor 8 (IRF8), and interleukin-18, displayed elevated expression in the lacrimal samples from both the diagnosed and suspected groups (**Figure 6B**). This suggests an enhanced activity of the TLR signaling pathway in the context of this disease, which may be closely associated with the molecular pathogenesis of IgG4-ROD.

### 3.7 Validation of potential molecular markers for IgG4-ROD

**Figure 7:**
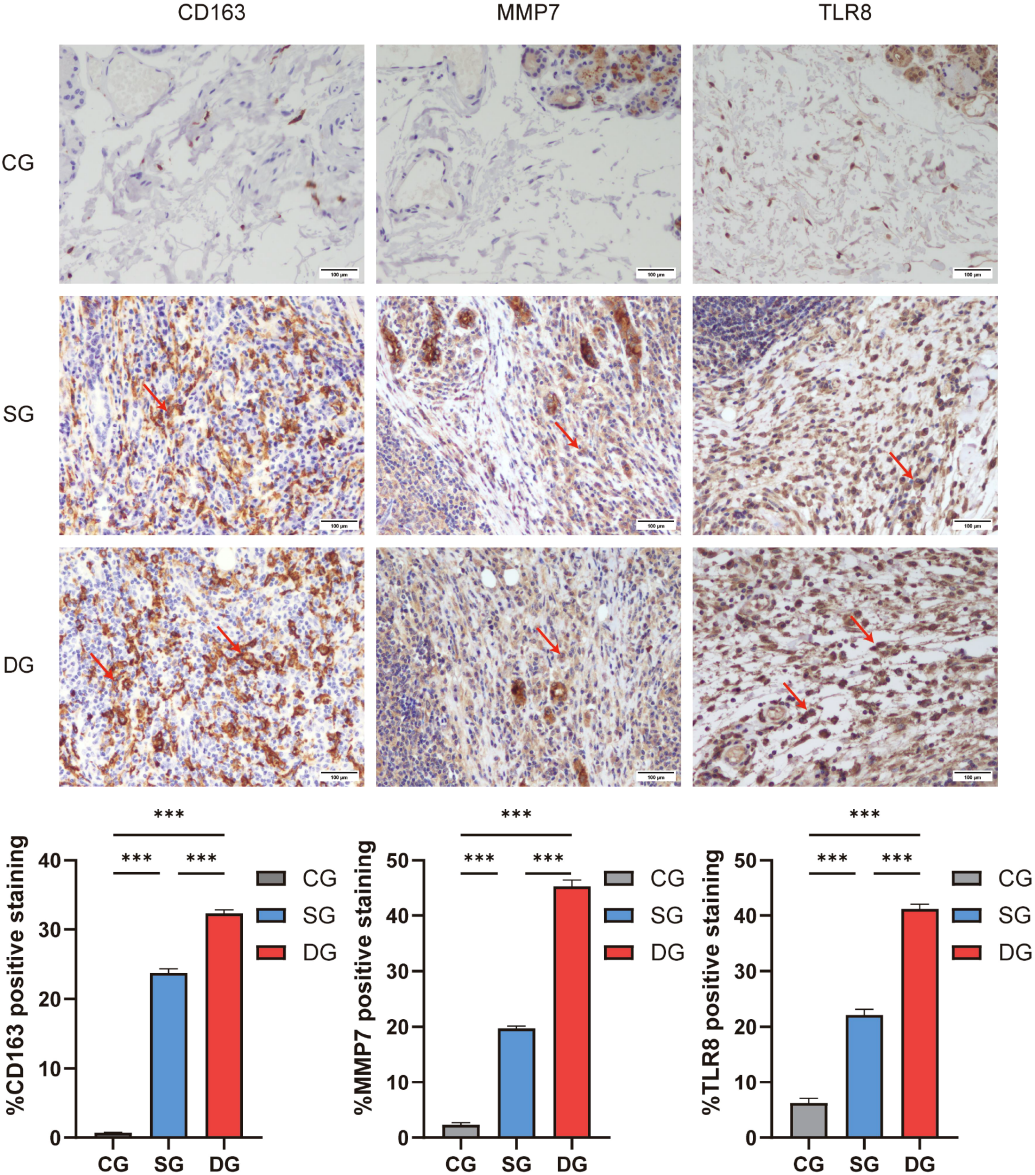
Immunohistochemical staining analysis of potential molecular markers for IgG4-ROD. We verified the expressions of CD163, MMP7 and TLR8 in the lacrimal gland tissues of the suspected group, the confirmed group and the control group of IgG4-ROD through immunohistochemical staining. Positively expressed cells were stained brown, as indicated by the red arrows. The expression level, determined by the percentage of positive signal area, was quantified and visualized in column graphs. Compared with the control group, CD163 showed strong positive expression in the lacrimal gland tissues of both the confirmed and suspected IgG4-ROD groups, especially in macrophages. MMP7 was diffusely and strongly positively expressed in fibroblasts, macrophages and plasma cells in the lacrimal gland tissues of the confirmed group and the suspected group. TLR8 was diffusely and strongly positively expressed in fibroblasts, macrophages, dendritic cells and plasma cells in the lacrimal gland tissues of the confirmed group and the suspected group. Scale bar: 100μm; DG, Diagnosed group; SG, Suspected group; CG, Control group. *** denotes very statistically significant.

Through the analysis of proteomic data, we found that the expression levels of CD163 and MMP7 were significantly increased in patients with confirmed IgG4-ROD, suggesting that they might be markers for the progression of fibrosis in the lacrimal gland tissue. In addition, the protein expression of TLR8 showed an up-regulated trend in the lacrimal gland samples of both the confirmed group and the suspected group, indicating that it plays an important role in the molecular mechanism of the disease progression of IgG4-ROD. Therefore, we further verified CD163, MMP7 and TLR8 in the lacrimal gland samples of the confirmed group, the suspected group and the control group by immunohistochemical staining. As shown in **Figure 7**, CD163 was characteristically highly expressed in the lacrimal gland tissues of both the confirmed and suspected IgG4-ROD groups, especially with strong positive expression in macrophages. MMP7 was diffusely and strongly positively expressed in fibroblasts, macrophages and plasma cells in the lacrimal gland tissues of the confirmed group and the suspected group, while it was negative or weakly expressed in the lacrimal gland tissues of the control group. Compared with the control group, TLR8 was diffuse.

ely and strongly positively expressed in fibroblasts, macrophages, dendritic cells and plasma cells in the lacrimal gland tissues of the confirmed and suspected IgG4-ROD groups. The results of immunohistochemical staining were consistent with our proteomic data analysis. In the lacrimal gland tissues of the confirmed and suspected IgG4-ROD groups, the protein expression levels of CD163, MMP7 and TLR8 were significantly higher than those in the control group, suggesting that CD163, MMP7 and TLR8 may serve as molecular markers to assist in the diagnosis of IgG4-ROD.

## 4 Discussion

This study innovatively conducts a case-control study on IgG4-ROD. For the first time, high-throughput proteomics technology is applied to analyze the protein expression profiles of lacrimal gland samples from IgG4-ROD patients with different disease severities. Through a comprehensive comparison of the protein expression profiles of normal lacrimal glands, lacrimal glands of patients suspected of having IgG4-ROD, and those of diagnosed patients, this study provides research evidence for exploring diagnostic biomarkers of IgG4-ROD. Meanwhile, it also paves the way for elucidating the molecular pathological mechanisms of the disease and developing molecular-targeted therapies.

Although IgG4 levels are typically elevated in the serum of patients with IgG4-related disease (IgG4-RD), this alteration is not sufficiently specific to serve as a foundation for early diagnosis. Currently, histopathology remains a crucial diagnostic criterion for IgG4-RD, with the number of IgG4^+^ plasma cells serving as pivotal evidence for diagnosis. The 2011 comprehensive diagnostic criteria for IgG4-RD already incorporated the number of IgG4^+^ plasma cells and the ratio of IgG4^+^/IgG^+^ plasma cells as components of the comprehensive diagnostic criteria for the disease (18). However, a substantial number of IgG4^+^ plasma cells are also present in other diseases, including non-small cell lung cancer, inflammatory pseudotumors of the lung, and pancreatic cancer (20–22). In this study, we noticed a progressive aggravation of clinical symptoms, imaging findings, and pathological changes from the suspected group to the diagnosed group. In comparison with the control group, the differential expression protein profiles (encompassing types of differentially expressed proteins and trends in upregulation and downregulation) of lacrimal gland samples from the suspected and diagnosed groups were highly similar. Moreover, functional enrichment analysis demonstrated that proteins were enriched in analogous signaling pathways, suggesting that patients in the diagnosed and suspected groups might belong to the same cohort. In clinical practice, some patients may display clinical symptoms and signs of IgG4-ROD but fail to fulfill the serological and pathological criteria, thereby hindering a definitive diagnosis. Nevertheless, protein expression changes might have already occurred in the lacrimal gland samples of these patients, and proteomic analysis should be taken into account. By utilizing high-throughput proteomics, we can compare the lacrimal gland protein expression profiles of these patients with those of diagnosed IgG4-ROD patients, enabling real-time monitoring of key protein expression changes in lacrimal gland tissues. Through precisely identifying the differential protein types, quantifying the dynamic trends in protein expression levels, and investigating the activation status of key signaling pathways, we can enhance the accuracy and timeliness of disease diagnosis, thereby providing timely diagnosis and intervention to prevent disease progression. Therefore, we believe that proteomic analysis has significant advantages in the molecular diagnosis of IgG4-ROD and may provide supportive evidence for suspected cases of IgG4-ROD.

Further comparison of protein expression levels between the diagnosed and suspected groups revealed that proteins downregulated in the diagnosed group were significantly enriched in elastin fiber-related signaling pathways. A reduction in elastin fibers is a characteristic of tissue fibrosis, indicating more severe fibrosis in the lacrimal gland tissue of the diagnosed group compared to the suspected group. Additionally, we examined the expression trends of proteins related to lacrimal gland secretion function. Compared with the control group, the expression levels of proteins involved in vesicle synthesis and transport in the lacrimal tissue of both the diagnosed and suspected groups exhibited a significant decrease, indicating impaired lacrimal secretion function. In summary, the diagnosed group displayed the most severe fibrosis and the greatest impairment of lacrimal secretion function, suggesting considerable lacrimal gland suggesting considerable lacrimal gland dysfunction in IgG4-ROD patients at the diagnosed stage.

This study, through proteomic analysis, provides the first demonstration of a synergistic mechanism between TLR8-mediated innate immune activation and Th2/Th17-imbalanced adaptive immune responses in IgG4-ROD. Our results reveal significant autoimmune dysregulation in the lacrimal gland tissues of IgG4-ROD patients. Proteomic data demonstrate marked upregulation of TLR8 signaling pathway-related proteins in both diagnosed and suspected IgG4-ROD patients. Meanwhile, the immunohistochemical staining experiment also confirmed this result. We propose that this pathway promotes fibroblast proliferation and M2 macrophage polarization through IRAK4-dependent NF-κB activation. In the pancreatic tissue of patients with IgG4-related autoimmune pancreatitis, eosinophils expressing Toll-like receptors (TLR2 and/or TLR4) are prominently infiltrated (23). Notably, previous studies have confirmed that CD163^+^ M2 macrophages expressing TLR7 can secrete interleukin-33, interleukin-1β, and TGF-β via the TLR7/IRAK4/NF-κB signaling pathway, promoting the activation and proliferation of fibroblasts in patients with IgG4-related sialadenitis (24). Toll-like receptors regulate innate immune responses and indirectly participate in autoimmune reactions, making them potential therapeutic targets for autoimmune connective tissue diseases (25). Our research findings corroborate these academic perspectives. Importantly, aberrant activation of TLR family members (particularly TLR2/4/7/8) may play a crucial role in autoimmune fibrosis by modulating the function of innate immune cells, including M2 macrophages, eosinophils, and plasmacytoid dendritic cells (DCs) (26–28). Although TLR7 and TLR8 share common downstream signaling cascades, their expression patterns and functional inclinations differ. TLR8 is distinctly expressed in neutrophils, monocytes, and myeloid dendritic cells. Moreover, upon activation, TLR8 preferentially promotes the NF-κB signaling pathway and the production of inflammatory cytokines (29, 30). Therefore, we infer that the TLR8/IRAK4/NF-κB signaling pathway may be involved in the molecular mechanism of IgG4-ROD by promoting fibrosis in the affected tissues. Our GO and KEGG database analyses revealed that differentially expressed proteins in both the diagnosed and suspected groups were significantly enriched in adaptive immune-related signaling pathways, including T cell activation, Th1 and Th2 differentiation, and IL-17-related signaling pathways. This finding aligns with multiple clinical observations. Miyake et al. observed that, in patients with Mikulicz’s disease, the Th1/Th2 balance in peripheral CD4^+^ T cells was altered, with an increase in Th2 cytokine expression (31). The alteration of the Th1/Th2 balance is linked to Th17 cells, a CD4^+^ Th cell subset characterized by the production of IL-17 (32). Maehara et al. found that, compared to normal individuals and patients with SS, patients with IgG4-RD had an increased number of Th2 and Th17 cells in peripheral blood, suggesting a biased peripheral T cell profile in IgG4-RD with increased Th2 and Th17 cell expression (33). Nirula et al. reported that IgG4-RD is often associated with a history of allergic conditions, such as bronchial asthma and allergic rhinitis, and that eosinophilia and elevated serum IgE levels are common in these patients (34). In allergic reactions, specific Th2 cytokines, such as IL-4 and IL-13, play an important role in promoting B cells to secrete IgG4 and IgE. Nakashima et al. suggested that Th2 immune responses may contribute to the pathogenesis of IgG4-related interstitial nephritis (35). This study proposes that TLR8-mediated innate immune activation forms a positive feedback loop with Th2/Th17 adaptive immune responses. TLR8 signaling pathway promotes inflammatory microenvironment formation through NF-κB, while Th2/Th17 cytokines further sustain the activated state of innate immune cells. This immune dysregulation ultimately leads to the characteristic pathological changes of tissue fibrosis and IgG4-positive plasma cell infiltration. These findings provide a potential theoretical basis for precision therapeutic strategies targeting the TLR8 signaling pathway and Th2/Th17 cytokines.

Through proteomic analysis, we observed that, in comparison with the control group, the diagnosed group exhibited a significant upregulation of several secreted proteins in their lacrimal gland samples. Secreted proteins reflect intercellular communication and the pathological state of tissues, rendering them easily detectable and suitable for screening. These proteins may serve as potential molecular biomarkers and therapeutic targets, facilitating the early diagnosis and intervention of IgG4-ROD. In our study, MMP7 was the most significantly upregulated secreted protein and plays a key role in tissue fibrosis. MMP7 is a member of the matrix metalloproteinase (MMP) family, primarily promoting fibrosis by degrading extracellular matrix (ECM) components, such as collagen III, IV, and fibronectin. In renal fibrosis, MMP7 is overexpressed, degrading type IV collagen and disrupting the integrity of the glomerular basement membrane, promoting inflammatory cell infiltration and creating conditions for the proliferation of fibrosis-associated cells. Once fibronectin is degraded by MMP7, it interferes with normal cell-ECM interactions, promoting fibroblast activation, proliferation, and migration, thus accelerating the development of fibrosis (36). MMP7 also activates TGF-β, which further promotes fibrosis. Activated TGF-β induces fibroblasts to synthesize more ECM components and differentiate into myofibroblasts, thereby increasing ECM deposition and driving tissue fibrosis (37, 38). Additionally, TGF-β stimulation induces epithelial-to-mesenchymal transition (EMT) in renal tubular epithelial cells, an important process in renal interstitial fibrosis (39). Studies have shown that the chronic progressive upregulation of β-catenin is a common pathological feature of various chronic kidney diseases with fibrosis, and MMP7-mediated E-cadherin degradation leads to the release and activation of β-catenin, which translocates to the nucleus. The released β-catenin translocates to the nucleus, driving the expression of target genes such as MMP7, thus promoting the occurrence of EMT and advancing fibrosis (38, 40). Chen et al. found that elevated MMP7 expression levels indicate active interstitial lesions in the lung and can serve as an early diagnostic marker for idiopathic pulmonary fibrosis, as well as an important indicator for evaluating disease progression (41). CD163, a member of the scavenger receptor family, was significantly upregulated in our study as well. CD163 is primarily expressed on the surface of monocyte-macrophages and plays a crucial role in tissue fibrosis. In the early stages of fibrosis, tissue damage triggers an inflammatory response that attracts monocytes to the lesion site, where they differentiate into macrophages. During this process, the expression of CD163 on these macrophages is significantly upregulated. CD163 specifically binds to hemoglobin-haptoglobin complexes (Hb-Hp) and internalizes them via receptor-mediated endocytosis, a process that plays a crucial role in regulating the inflammatory microenvironment (42, 43). As fibrosis progresses, CD163 actively participates in the polarization of macrophages. Under the influence of cytokines such as IL-4 and interleukin-13 (IL-13), CD163-expressing macrophages tend to polarize into M2 macrophages that secrete large amounts of TGF-β, a key driver of the fibrosis process, which further promotes tissue fibrosis (44). Additionally, M2 macrophages release platelet-derived growth factor, which stimulates the proliferation and migration of fibroblasts and smooth muscle cells, exacerbating the abnormal accumulation and activation of fibrosis-related cells in tissues and worsening the extent of fibrosis (45). Notably, the release of CD163 by M2 macrophages correlates with the degree of tissue fibrosis. Ragab et al. proposed that CD163 could serve as a molecular marker for diagnosing non-alcoholic fatty liver disease (46). Furthermore, we observed that periostin (POSTN) expression was significantly increased in the lacrimal gland tissues of patients diagnosed with IgG4-ROD, consistent with the findings of Nobuo Ohta et al. (47). Upon tissue damage, POSTN interacts with cell surface integrin receptors, activating signaling pathways that promote fibroblast migration and activation. Masamitsu Hara et al. observed that POSTN enhanced the migration of fibroblasts to injured muscle tissues, exacerbating fibrotic scar formation (48). POSTN also participates in the formation and remodeling of collagen fibers and ECM, promoting fibroblast transformation into myofibroblasts, which accelerates the fibrosis process (49). These results suggest that POSTN may serve as a molecular marker for reflecting fibrosis progression in the lacrimal gland tissues of IgG4-ROD patients. The aforementioned secreted proteins may serve as molecular biomarkers for the early diagnosis of IgG4-ROD, although further experimental validation is required.

Furthermore, compared with the suspected group, we observed that proteins with elevated expression levels in the lacrimal gland tissue of the confirmed group were enriched in biological processes related to platelet activation. While most studies have focused on the pathogenic roles of immune cells, such as lymphocytes and macrophages, Herter et al. found that platelets play an immune-regulatory role in various immune-inflammatory conditions (50). Research by Zhu et al. indicated that platelets initially promote the activation of Th1, Th17, and Treg cells and subsequently inhibit the immune responses of Th1 and Th17 cells (51). Platelets express receptors such as FcγRIIA, TLR4, and TLR9, which receive stimuli from the microenvironment and respond accordingly. Platelet granules contain various growth factors, chemokines, and pro-inflammatory factors, including TGF-β, epidermal growth factor and CXCL12, all of which contribute to platelet-mediated regulation in immune-inflammatory processes (52). Therefore, future research should further explore the immune functions of platelets in IgG4-ROD.

Nevertheless, this study has limitations. The sample size is small, and the proteomic methodology employed may be subject to inherent technical biases, underscoring the need for future validation through large-scale, multi-center investigations. The proposed molecular mechanisms will require functional verification in a suitable IgG4-ROD animal model once such a model becomes available. Furthermore, while the current work focused exclusively on proteomic profiling, future studies integrating transcriptomic, lipidomic, metabolomic, and glycomic data would offer a more comprehensive understanding of the disease pathogenesis.

## 5 Conclusion

To the best of our knowledge, this study is the first to perform high-throughput proteomic analysis of lacrimal gland samples from patients with autoimmune IgG4-ROD of varying severity. We analyzed the clinical characteristics, imaging findings, and histopathological features of IgG4-ROD patients, suggesting that the diagnosed and suspected groups belong to the same patient cohort. Proteomic analysis revealed a more advanced degree of fibrosis in the diagnosed group compared to the suspected group, reflecting the progression of the disease at different stages of severity. We identified significantly elevated levels of MMP7, CD163, and POSTN in the lacrimal gland tissues of IgG4-ROD diagnosed patients, indicating that these proteins may serve as biomarkers for the progression of lacrimal gland fibrosis and could potentially be utilized as molecular markers for early diagnosis and therapeutic targets in IgG4-ROD. Additionally, proteomic analysis identified signaling pathways potentially involved in the pathogenesis of the disease. We hypothesize that the TLR8/IRAK4/NF-κB signaling pathway may contribute to the molecular mechanisms of IgG4-ROD by promoting fibrosis in the affected tissues and cooperating with the adaptive immune response of Th2/Th17 imbalance. Based on these findings, we propose that proteomic technology could serve as a supportive tool for the diagnosis of IgG4-ROD. Our research also provides insights into the development of IgG4-ROD and explores potential molecular mechanisms underlying the disease, laying the foundation for the future development of molecular diagnostics and targeted therapies for IgG4-ROD.

## Data Availability

Data generated during the current study are available from the corresponding author upon reasonable request.

## Glossary

ANOVA: Analysis of Variance
BCA: Bicinchoninic Acid Assay
CTL: cytotoxic T lymphocyte
CYCS: cytochrome C, somatic
DIA: data-independent acquisition
ECM: Extracellular Matrix
EMT: Epithelial-to-Mesenchymal Transition
FDR: False Discovery Rate
GO: Gene Ontology
HE: hematoxylin and eosin
IgG4-RD: IgG4-related disease
IgG4-ROD: IgG4-related ophthalmic disease
IGHG4: immunoglobulin heavy constant gamma 4
IL-4: interleukin-4
IL-13: interleukin-13
IL-17: interleukin-17
IL4I1: interleukin-4 Induced 1
IRAK4: interleukin-1 receptor-associated kinase 4
IRF3: interferon regulatory factor 3
IRF7: interferon regulatory factor 7
IRF8: interferon regulatory factor 8
KEGG: Kyoto Encyclopedia of Genes and Genomes
MMP7: Matrix Metalloproteinase 7
MS: Mass Spectrometry
Myd88: myeloid differentiation primary response 88
NF-κB: nuclear factor-kappa B
PCA: principal component analysis
PI3K-AKT: phosphatidylinositol 3 kinase-protein kinase B
POSTN: Periostin
ROS: reactive oxygen species
SABC: Strept Avidin-Biotin Complex
SS: Sjögren’s syndrome
TGF-β: transforming growth factor-beta
Th1: T helper 1
Th2: T helper 2
Th17: T helper 17
TLR: Toll-like receptor
TRAF3IP3: TRAF3 Interacting Protein 3
